# Dermal immune responses against *Psoroptes ovis* in two cattle breeds and effects of anti-inflammatory dexamethasone treatment on the development of psoroptic mange

**DOI:** 10.1186/s13567-020-00874-x

**Published:** 2021-01-04

**Authors:** Zhenzhen Chen, Edwin Claerebout, Koen Chiers, Mathilde Pas, Bart Pardon, Wouter van Mol, Stijn Casaert, Nathalie De Wilde, Luc Duchateau, Peter Geldhof

**Affiliations:** 1 Laboratory of Parasitology, Faculty of Veterinary Medicine, Ghent University, Salisburylaan 133, 9820 Merelbeke, Belgium; 2 Department of Pathology, Bacteriology and Poultry Diseases, Faculty of Veterinary Medicine, Ghent University, Salisburylaan 133, 9820 Merelbeke, Belgium; 3 Department of Large Animal Internal Medicine, Faculty of Veterinary Medicine, Ghent University, Salisburylaan 133, 9820 Merelbeke, Belgium; 4 Biometrics Research Center, Faculty of Veterinary Medicine, Ghent University, Salisburylaan 133, 9820 Merelbeke, Belgium

**Keywords:** *Psoroptes ovis*, immune responses, cattle breeds, anti-inflammatory treatment

## Abstract

Psoroptic mange is a common disease of livestock, caused by *Psoroptes ovis*. Compared to Holstein–Friesian (HF) cattle, the Belgian Blue (BB) cattle breed is highly susceptible to the infestation. However, the mechanism for this difference is still unclear. To determine the factors responsible for this breed susceptibility, the immune response to *P. ovis* was studied in experimentally infested BB and HF cattle, using clinical signs, histology, immunohistochemical profiling and gene expression analysis of skin biopsies. The mite numbers and lesion area of BB cattle were greater than in HF during the whole study period. Significant influxes of eosinophils in the epidermis and dermis were detected in comparison with the pre-infestation samples in both breeds, with significantly higher eosinophils in BB at 6 weeks post infestation (wpi). Mast cell numbers were unaffected at all stages of infestation in HF, but were significantly elevated relative to pre-infestation in BB cattle at 2 and 6 wpi. The more pronounced cutaneous eosinophilia and higher IL-4 levels at 6 wpi in BB cattle suggest that a Th2-type immune response is underlying the higher susceptibility of the BB breed. In naturally infested BB cattle, development of the psoroptic mange lesions and eosinophils and CD3+ T cell areas were severely depressed after anti-inflammatory treatment with dexamethasone. Together, these results suggest that a stronger Th2-type immune response to *P. ovis* causes the skin lesions in psoroptic mange in BB cattle and that local anti-inflammatory treatment could potentially be an alternative to control the pathology caused by this parasite.

## Introduction

Psoroptic mange is a severe disease, which reduces animal welfare and causes financial losses due to performance loss and treatment costs, especially in the sheep and in the beef industry [1–3]. This disease is caused by a non-burrowing ectoparasite, *Psoroptes ovis*, which lives on the skin surface. The mouthparts of these mites are adapted to the consumption of serous exudate, lymph and red blood cells from the host skin surface [4]. Meanwhile, the mites abrade the *stratum corneum* and deposit allergens (such as faecal pellets), which cause skin irritation, intense pruritus, and severe allergic dermatitis in livestock [5]. The cutaneous inflammatory response developed by hosts in response to the mites is a serious threat to epidermal integrity [6–8]. The damage in the epidermis is considered as promoting the hypersensitivity reaction in the host to control the mite infestation [9, 10]. Previous work in sheep demonstrated that a reduction of lesion development and mite numbers was caused by suppressing the host immune response [11]. However, the relationship between lesion development and immunosuppression in cattle has not been clarified.

Generally, the development of clinical signs in cattle starts at 1 week post infestation [10]. Next, a rapid growth phase sets in with a sharp increase in mite numbers and active lesions that are mainly found at the withers, back and the tail base, but can eventually cover most of the body [3, 12–14]. During this process, pruritus becomes more intense due to the host allergic reaction to the mites, leading to self-trauma behaviour, such as licking and rubbing. Although these actions will make the affected animals comfortable to some degree, mechanical skin abrasion can cause hair loss, skin damage and bleeding wounds [13–15]. All these factors will intensify the local intradermal inflammation, which leads to increased serum extravasation, creating the perfect microclimate for mites to survive. This ideal environment will in turn translate in a further growth of both mite population and skin lesions [10]. Subsequently, the lesion size and mite numbers decrease and, eventually, the clinical signs disappear and the mites are eliminated [2]. However, whether or not animals fully recover from the disease depends on different factors, such as the cattle breed [2, 16]. For instance, natural recovery in Holstein Friesian (HF) cattle is more frequent compared to Belgian Blue (BB) cattle [13].

Previous research suggested a high susceptibility to *P. ovis* infestation in BB cattle under field conditions compared to HF cattle [2, 17]. Preliminary data suggests that a difference in immune response may contribute to the different breed susceptibility. Previous work revealed that numerous degranulating mast cells and neutrophils were recruited in the skin of Hereford heifer calves in response to *P. ovis* infestation [18], but mast cells did not significantly differ between naturally infested BB and HF cattle [19]. Interestingly, after intradermal injection of *Psoroptes cuniculi* antigen in cattle of different breeds, an immediate hypersensitivity reaction was observed after 1 h in HF and BB animals; whereas only in BB cattle a delayed hypersensitivity reaction was recorded after 72 h [2, 20]. Moreover, Sarre et al. [19] demonstrated that the up-regulation of IL-17 in the skin of BB cattle was associated with a higher sensitivity to *P. ovis* compared to HF cattle. However, this study was performed in naturally infested animals, which made it impossible to control infestation levels and to determine temporal dynamics of immune responses.

Therefore, in this study, we aimed to compare the development of lesions and the immune response against *P. ovis* between artificially infested BB and HF cattle, and to investigate the relationship between the inflammatory reaction of the host and lesion development, using an anti-inflammatory dose of dexamethasone in BB cattle. Results were compared with published data from sheep [6, 21] and Hereford heifer calves [18], in order to gain insight into critical factors which may be involved in the susceptibility of the BB cattle breed and the relative resistance of the HF cattle breed.

## Materials and methods

### Animal study one: development of psoroptic mange in Belgian Blue and Holstein–Friesian cattle

Nine healthy BB calves and six healthy HF calves (6–7 months old) were included. All calves were free of mite and lice infestation as determined by thorough body inspection and examination of skin scrapings prior to the experiment. All animals were individually stanchioned, with a metal frame around the neck to restrict grooming. *P. ovis* mites (nymphs and adults) were isolated from naturally infested BB cattle. An area of the left side of the withers (15 cm diameter circle) of each animal was shaved with electric clippers, and approximately 400 ~ 500 mites were placed directly onto the skin of each animal. Mites were prevented from escaping with either a filter paper that was kept in place by tying a rubber band around the animal’s chest (n = 4 BB) or with syringes that were cut at the base and glued (super glue, Loctite, Belgium) onto the skin (5 BB and 6 HF), with each syringe containing ~ 100 mites.

Skin biopsies were collected from the infestation site on day 0 (before infestation) and thereafter from the edge of active lesions at weekly intervals from 2 until 6 weeks after infestation. Two skin biopsies were obtained using a disposable 4 mm biopsy punch (pfm Medical, Germany). Prior to biopsy, the site was subcutaneously injected with a local anaesthetic (3–4 mL, 4% procaine hydrochloride and 0.0036% adrenaline tartras, KELA, S.C.-epidural, Belgium). One biopsy was snap-frozen in liquid nitrogen and stored at − 80 °C until RNA extraction was performed. The other one was stored for 10 h in 4% formaldehyde and paraffin-embedded for histology and immunohistochemistry.

The clinical degree of infestation was determined weekly by calculating the percentage of infested body surface (clinical index, CI) for each animal, based on the method of Guillot [22], from day 0 until week 6 p.i. Briefly, all the lesions on the surface of an animal’s body were recorded in the grid and then the percentage body surface affected by lesions was calculated. At week 6, *P. ovis* mite numbers in active lesions were counted. Skin scrapings were collected from the edges of active lesions or, if lesions regressed during the study, from the area where active lesions were at the study commencement, according to the guidelines of the World Association for the Advancement of Veterinary Parasitology [23]. In total three lesion sites per animal were sampled by scraping an area of 9 cm^2^ per lesion. Samples were examined within 24 h of collection to identify and count *P. ovis* mites under the microscope (400× magnification). At the termination of the experiment, all animals were treated topically, twice with a 1-week interval, with amitraz (Taktic®) at the recommended dose.

### Animal study two: effect of anti-inflammatory treatment on development of psoroptic mange

Fourteen Belgian Blue calves (1–2 years old) naturally infested with *P. ovis* mites were included in the animal study. Skin scrapings were collected from each animal for mite counts and mite identification on day-7. The CI was determined for each animal by recording the skin lesions (on both sides of the animal) on a silhouette [22]. Animals were randomly assigned to treatment and control group using CI as stratification factor.

On day 0, all animals in the treatment group were weighed and injected intramuscularly with dexamethasone (MSD Animal Health, Belgium) at a dose of 0.06 mg/kg body weight. Control animals were injected with the same volume of physiological saline (0.9%). On day 7 and day 14, the treatment was repeated.

All animals were followed for 4 weeks, whereby the CI was determined weekly for each animal as described above. Punch biopsies were taken on day 0, day 7 and day 28 from the edge of active lesions, following the administration of a local anaesthetic (3–4 mL 4% procaine hydrochloride and 0.0036% adrenaline tartrate, KELA, S.C.-epidural, Belgium). The 4 mm biopsy was immediately fixed in 4% formaldehyde and paraffin-embedded for histology and immunohistochemistry. At day-7 and day 28 post treatment, *P. ovis* mites in the active lesions were counted, as described above.

All animals were housed together in a pen on straw bedding, and were provided with corn silage, grass silage and water ad libitum and a daily ration of 1.5–2.0 kg concentrates per animal. All animals were checked weekly for any adverse reactions to the dexamethasone treatment by clinical examination [24] and by ultrasonography (Tringa Linear Vet, Esaote, the Netherlands) to detect (sub)clinical pneumonia. At the end of the animal study all animals were treated topically with amitraz with 2 weeks interval as described above.

### Histological and immunohistochemical examination of skin biopsies

Serial sections (4–5 μm) were cut from paraffin embedded biopsies, dewaxed and stained with haematoxylin and eosin (H&E) for the detection of eosinophils or with Giemsa staining for the detection of mast cells. The number of mast cells and eosinophils was determined at magnification of 400× using a LEICA light microscope. Therefore, ten photographs were analysed in different dermal layers for each section, i.e. 5 photographs were randomly taken from the epidermis/superficial dermis, and 5 from the deep dermis. Results were expressed as the number of cells per 10^5^ μm^2^ tissue surface. The investigator was blinded to the allocated group. An ocular micrometer was used to measure the width of epidermal layer. Five measurements were taken along transects along the skin surface. The thickness was expressed as the mean of 5 measurements at random sites.

Two sections were immunolabelled for CD3 (T cells) and CD20 (B cells) based on [19]. In short, skin tissue sections of 4 μm were mounted on APES-coated slides, blocked with H_2_O_2_ and stained with polyclonal rabbit anti-human CD3 (Dako, Belgium) or rabbit anti-human CD20 (Sigma-Aldrich, USA) antibodies. T- and B-cells were visualized by adding peroxidase labelled goat anti-rabbit antibodies (Dako, Belgium), diaminobenzidine tetrahydrochloride (DAB; Dako, Belgium) and by performing a counterstaining with haematoxylin. Pictures at magnification of 400× using a LEICA light microscope were randomly selected from the epidermis/superficial dermis and the deep dermis as described above. The area percentage of T-cells and B-cells was determined using the Colour Deconvolution plugin in ImageJ [25].

### Quantitative real-time polymerase chain reaction (PCR)

The TissueLyser II instrument (Qiagen) was used to disrupt and homogenize the frozen skin samples. Total RNA was extracted with the RNeasy mini kit (Qiagen) following the manufacturer’s protocol with on-column DNase digestion. RNA Quality Index (RQI) was assessed on the Experion automated electrophoresis system (Bio-Rad Laboratories) with the Experion RNA StdSence Analysis kit (Bio-Rad). RNA yield was assessed on a Nano-Drop 2000 spectrophotometer (Thermo Scientific). RNA samples with a RQI > 7.5 were considered to be of acceptable quality [26]. cDNA was obtained by reverse transcribing 100 ~ 150 ng/μL of purified total RNA using the iScript cDNA synthesis kit (Bio-rad). The cDNA was diluted 1:3 in dH_2_O and stored at − 20 °C.

A quantitative Real-Time PCR (qPCR) approach was used to measure the relative mRNA transcription levels of a selection of genes in the BB and HF calves’ skin. The sequences of all the primers that were used can be found in Table 1. qPCR and normalization of the data, based on the housekeeping genes *GAPDH* and *RPS29* for BB and HF, were performed as previously described [19]. Gene transcription levels were evaluated based on relative expression level between the different timepoints.

**Table 1:** Primer sequences and amplicon details for BB cattle and HF cattle qPCR

Gene	Accession number	Primer sequence
GAPDH	NM_001034034.1	F: ACCCAGAAGACTGTGGATGG
		R: CAACAGACACGTTGGGAGTG
RPS29	BC_102702	F: GGAGCCATCCGAGAAAATTCG
		R: CAACTTAATGAAGCCGATGTCCTT
IL-4	NM_173921.2	F: GCGGACTTGACAGGAATCTC
		R: TCAGCGTACTTGTGCTCGTC
IL-5	NM_173922.1	F: TGGTGGCAGAGACCTTGACA
		R: TTCCCATCACCTATCAGCAGAGT
IL-6	NM_173923.2	F: TCCTTGCTGCTTTCACACTC
		R: CACCCCAGGCAGACTACTTC
IL-8	NM_173925.2	F: GCTGGCTGTTGCTCTCTTGG
		R: GGGTGGAAAGGTGTGGAATGTG
IL-10	NM_174088.1	F: TGTATCCACTTGCCAACCAG
		R: CAGCAGAGACTGGGTCAACA
IL-13	NM_174089.1	F: GGTGGCCTCACCTCCCCAAG
		R: ATGACACTGCAGTTGGAGATGCTG
IL-17	NM_001008412.2	TGAGGACAAGAACTTCCCACAGCA
		TAATCGGTGGGCCTTCTGGAGTTT
IL-23A	NM_0012005688.1	F: CCCGTATCCAGTGTGAGGAT
		R: AGTATGGAGGCGTGAAGCTG
*IFN*-*γ*	NM_174086.1	F: TTCTTGAATGGCAGCTCTGA
		R: TTCTCTTCGGCTTTCTGAGG
Foxp3	NM_001045933.1	F: GACAGCACCCTTTCGACTGT
		R: CTCCAGAGATTGCACCACCT
Filaggrin	XM_010826841	F: GCCCAGTTCTAGACGCTGAC
		R: TCAAGCCAGTGACAGTGAGG
Involucrin	XM_005203832	F: AAGGTCTTGGGCCAGCACTTG
		R: GATGCTGGGTTGTAACTCCCCCCAC
Loricrin	NM_001113757	F: CAGTGGATCCGTCTGCCTGGGA
		R: CATGAGAGCGGTAAGCCCATCGAC

IL8 (CXCL8) designed based on bovine reference gene sequences from NCBI database. The other primers based on Sarre et al. [19].

### Statistical analysis

All analyses were done using GraphPad Prism 8 (GraphPad Software, San Diego, CA). First, comparing different time points with baseline within group or breed was done in the framework of rank-based repeated measures ANOVA, using Friedman’s multiple comparisons technique. Comparing the two groups or breeds at a particular time point was based on the Mann–Whitney U test using Bonferroni multiple comparisons.

## Results

### Lesion development and immune response in artificially infested cattle

**Figure 1:**
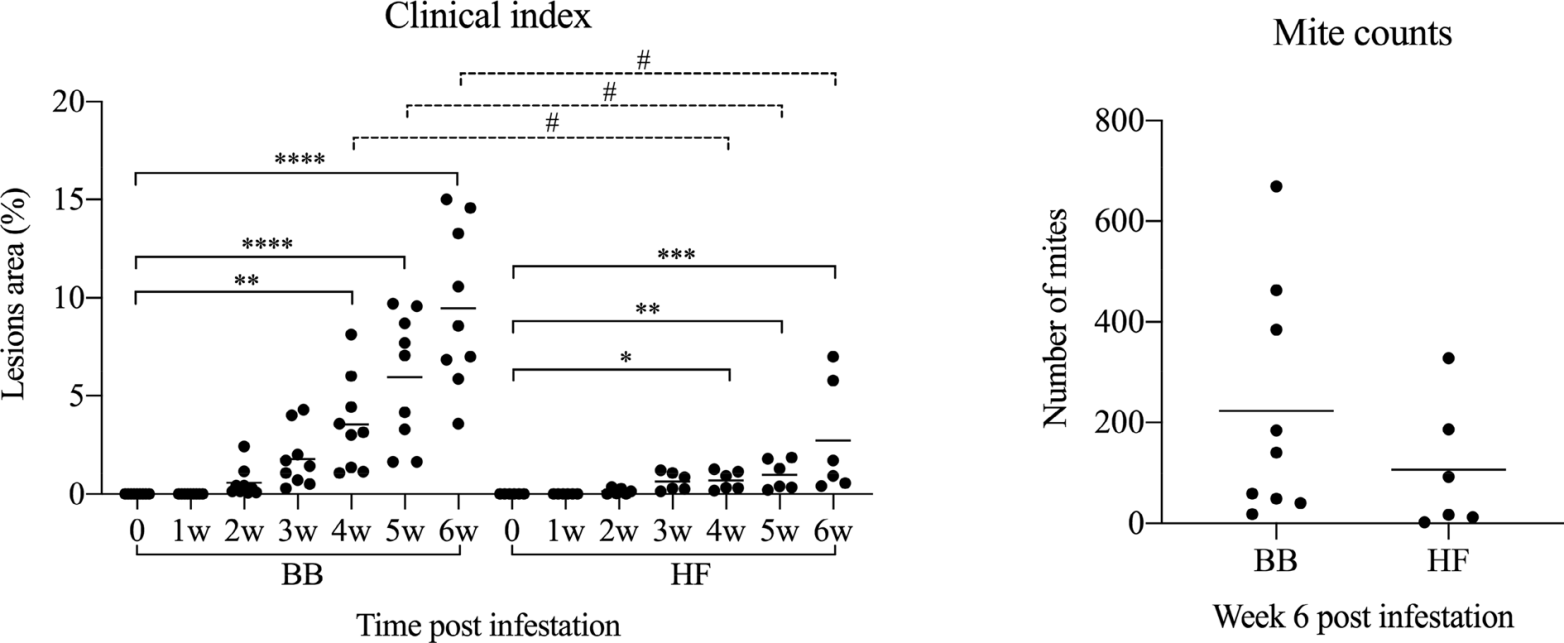
**The clinical indices and mite counts (mean and individual values) in Belgian Blue and Holstein–Friesian cattle after artificial infestation with** ***Psoroptes ovis***. Statistical significance within breed was assumed at *p < 0.05, **p < 0.01, ***p < 0.001, and ****p < 0.0001; Statistical significance between two breeds was assumed at ^#^p < 0.0083, and ^##^p < 0.0017.

**Figure 2:**
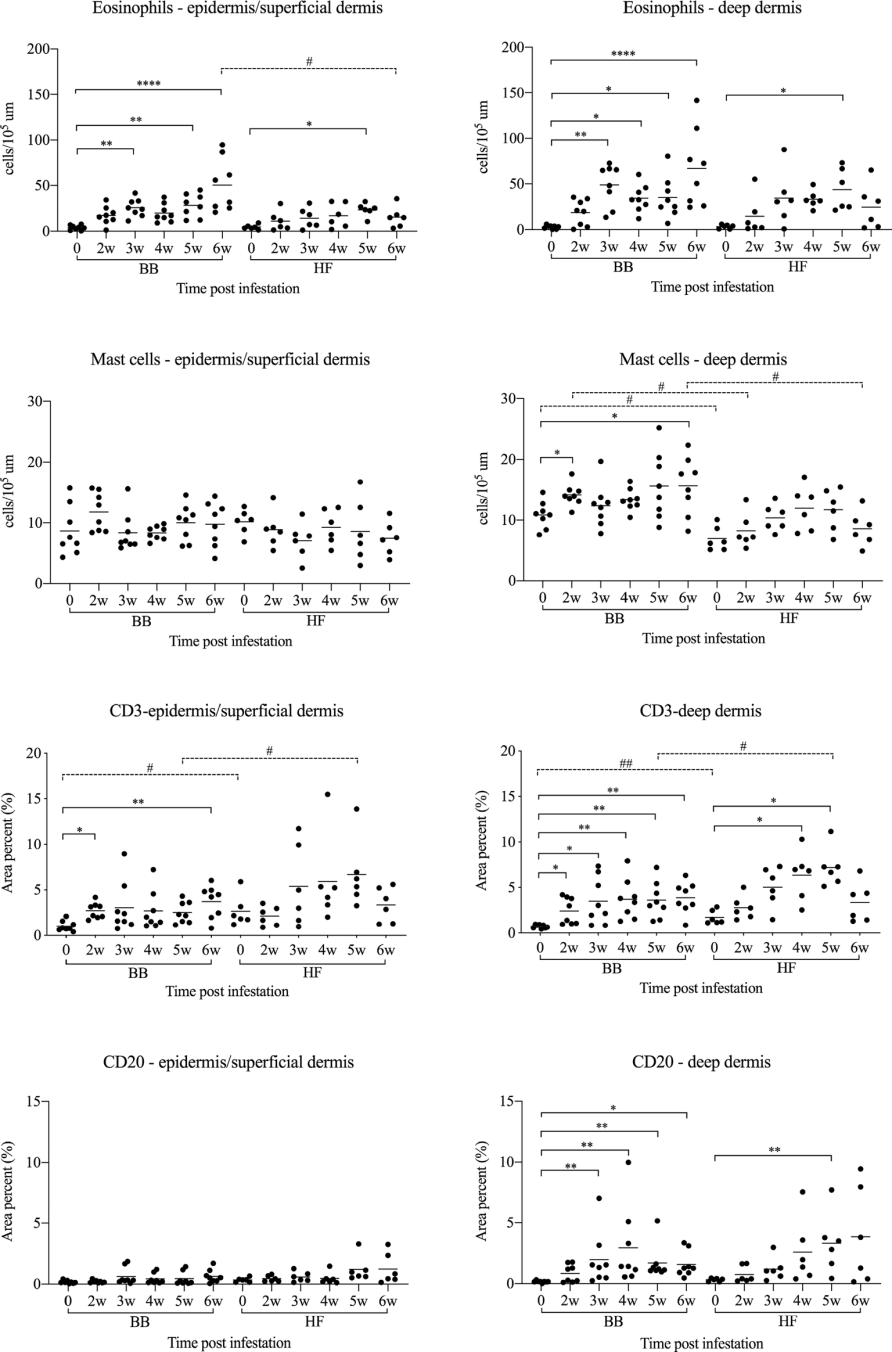
**Histological cell counts (mean and individual values) in the epidermis/superficial dermis and the deep dermis of skin biopsies in Belgian Blue and Holstein–Friesian cattle at different time points after artificial infestation with** ***Psoroptes ovis***. The results are presented as number of eosinophils and mast cells per 10^5^ μm^2^ and as area percent of CD3 + (T-cells) and CD20 + (B-cells). Statistical significance within breed was assumed at *p < 0.05, **p < 0.01, ***p < 0.001, and ****p < 0.0001; statistical significance between two breeds was assumed at ^#^p < 0.0083, and ^##^p < 0.0017. Only 8 BB cattle were included in the statistical analysis of the cell counts as from one animal missing values were obtained at 4wpi.

**Figure 3:**
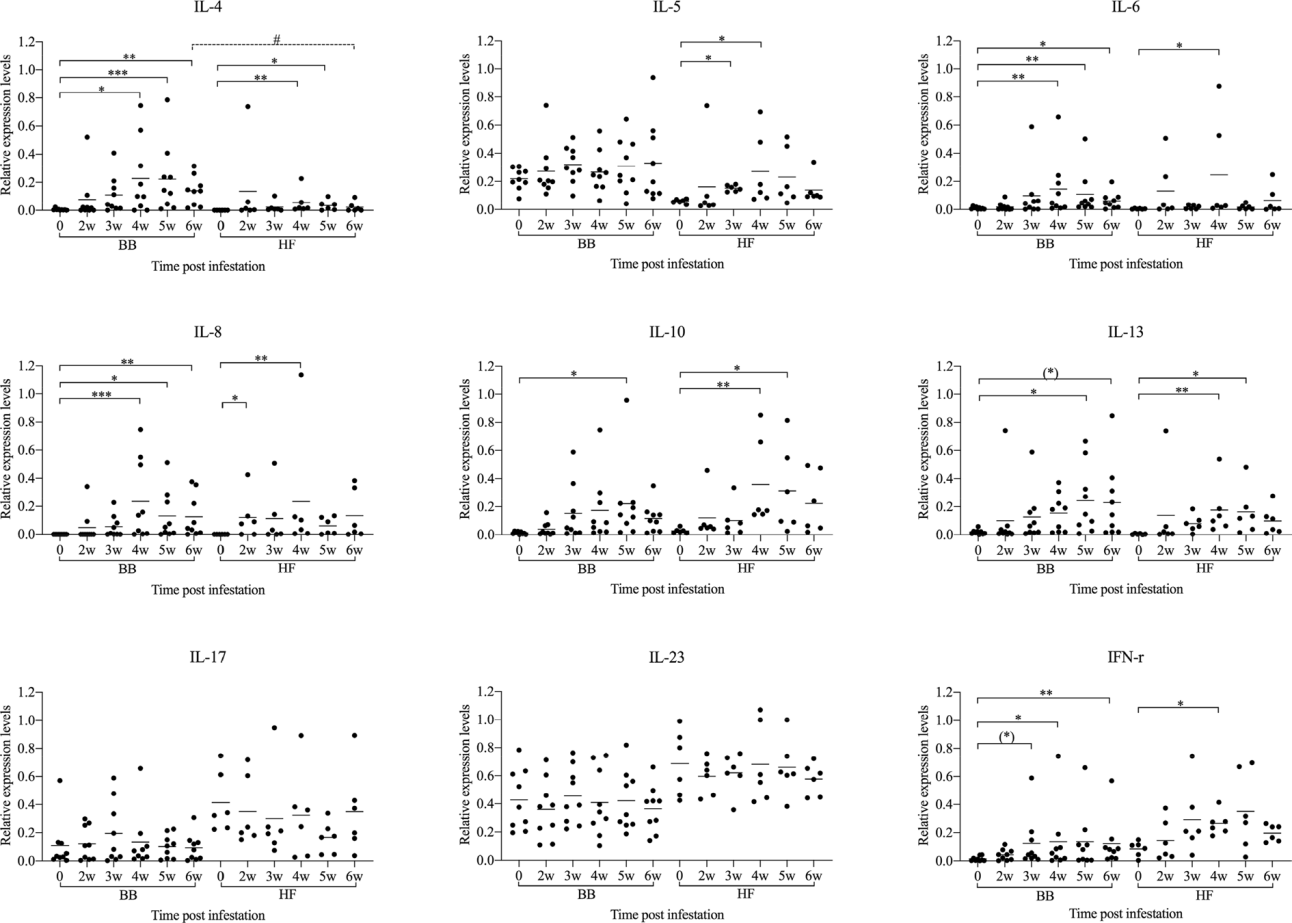
**The relative expression values of cytokines (mean and individual values) in Belgian Blue and Holstein–Friesian cattle at different time points after artificial infestation with** ***Psoroptes ovis***. Statistical significance within breed was assumed at ^(^*^)^p = 0.058, *p < 0.05, **p < 0.01, and ***p < 0.001; statistical significance between two breeds was assumed at ^#^p < 0.0083, and ^##^p < 0.0017.

**Figure 4:**
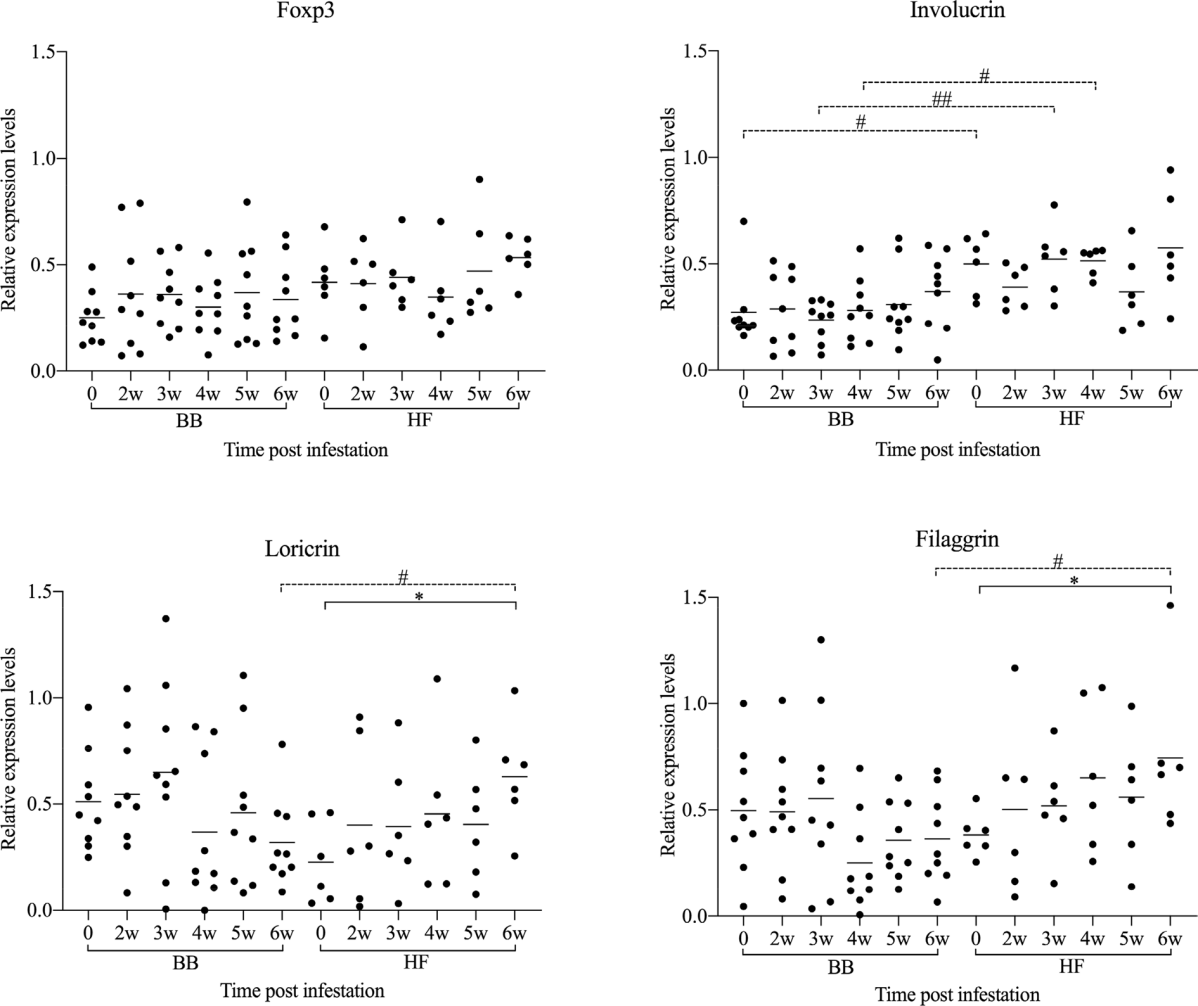
**The relative transcription levels of genes related to pathology (mean and individual values) in Belgian Blue and Holstein–Friesian cattle at different time points after artificial infestation with** ***Psoroptes ovis***. Statistical significance within breed was assumed at *p < 0.05; statistical significance between two breeds was assumed at ^#^p < 0.0083, and ^##^p < 0.0017.

Before infestation, no skin lesions were observed in the BB and HF cattle. Active lesions were first observed in both breeds at 2 weeks post infection (wpi). The CI significantly increased in both breeds from 4 wpi onwards (Figure 1) and at 6 wpi, both breeds showed typical clinical signs of psoroptic mange (Additional file 1). Major epidermal pathological changes of *P. ovis* infestation in both breeds included epidermal hyperplasia with marked acanthosis, diffuse hyperkeratosis, irregular rete ridge formation, apoptosis and transudation. Additional pathology in the dermis included oedema, vasculitis and dermal fibrosis (Additional files 2 and 3). Significant influxes of eosinophils, T-lymphocytes and B-lymphocytes in the epidermis/superficial dermis or the deep dermis were detected in comparison with the pre-infestation samples (Figure 2). Mast cell numbers appeared to be unaffected at all stages of infestation in the epidermis and superficial dermis, and were only significantly elevated relative to pre-infestation in the deep dermis of BB cattle (Figure 2). *P. ovis* infestation was associated with significantly increased transcription of several cytokines and genes related to skin pathology, including interleukin (IL)-4, IL-5 (in HF only), IL-6, IL-8, IL-10, IL-13, IFN-γ, loricrin (in HF only) and filaggrin (in HF only). No significant changes in IL-17, IL-23, Foxp3 and involucrin transcription were detected in both breeds (Figures 3 and 4), although there was a tendency towards a lower loricrin and filaggrin transcription in BB from 4 wpi onwards (Figure 4).

When both breeds were compared, the clinical index was significantly higher in BB cattle than in HF cattle at 4, 5 and 6 wpi. Although the mite numbers did not significantly differ between the two breeds, there was a trend for higher mite numbers in the BB animals (Figure 1). Compared with HF cattle, significantly higher eosinophil and mast cell infiltration was observed in BB cattle at 6 wpi in the epidermis/superficial dermis and the deep dermis, respectively (Figure 2). In contrast, a larger CD3-positive T-cell area was detected in HF cattle than in BB cattle in the epidermis/superficial dermis and the deep dermis at 5 wpi (Figure 2). Surprisingly, the CD3-positive T-cell area in HF cattle was already significantly higher than in BB cattle in the epidermis/superficial dermis and the deep dermis before infestation. Although a larger CD20-positive B-cell area was detected in the epidermis/superficial dermis and the deep dermis in HF cattle than in BB cattle, there was no significant difference between the two breeds. Compared to HF cattle, only the relative transcription levels of IL-4 were significant higher in BB cattle at 6 wpi (Figure 3). The transcription levels of loricrin and filaggrin were significantly higher in HF cattle than in BB cattle at 6 wpi, and the transcription levels of involucrin were significantly higher in HF cattle before infestation and at 3 and 4 wpi (Figure 4).

### Effect of anti-inflammatory treatment on development of psoroptic mange in BB cattle

**Figure 5:**
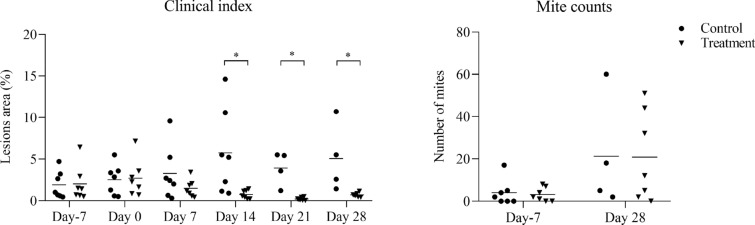
**The clinical index and mite counts (mean and individual values) in Belgian Blue cattle that were treated with dexamethasone on days 0, 7 and 14 (Treatment group) or physiological saline (Control group)**. Statistical significance was assumed at *p < 0.01 and **p < 0.002 for clinical index; statistical significance was assumed at *p < 0.05 for mite counts). On day 14, three animals in the control group were treated topically with amitraz at the recommended dose for animal welfare reasons.

Active lesions in the control animals steadily increased from day 0 onwards. On day 14, three animals in the control group were treated topically with amitraz at the recommended dose for animal welfare reasons. Compared to the control animals, there was a profound reduction in the development of the lesion area by 2 weeks after dexamethasone treatment (day 14), and this difference was maintained until termination of the study (day 28), when the CI was decreased by 90% compared to the control group. Mean mite counts were reduced by 56% in the treated group compared with the control animals on day 28, but the number of mites was not significantly different between the two groups (Figure 5).

**Figure 6:**
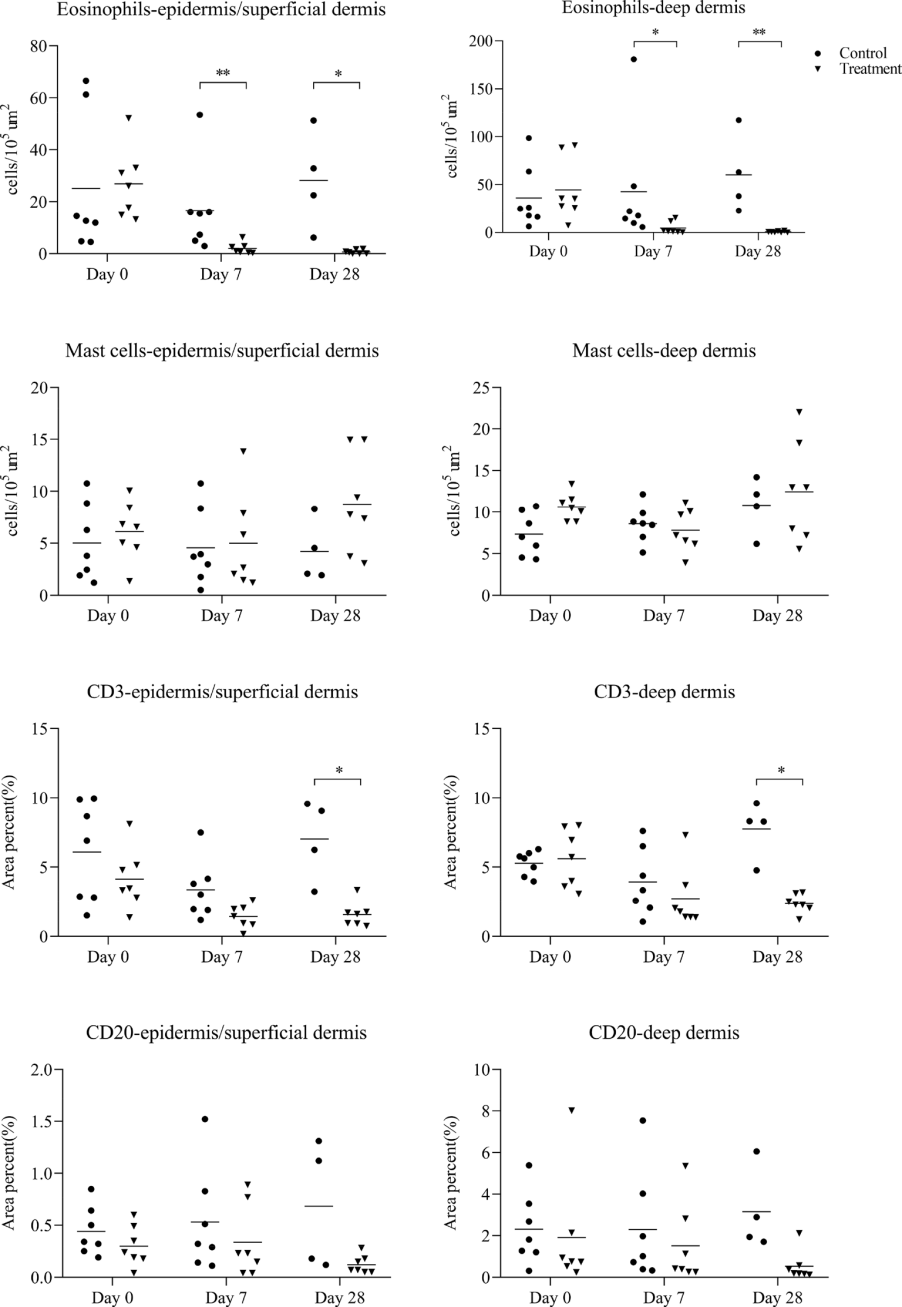
**Histological cell counts (mean and individual values) in the epidermis/superficial dermis and the deep dermis of skin biopsies in Belgian Blue cattle that were treated with dexamethasone on days 0, 7 and 14 (Treatment group) or with physiological saline (Control group).** The results are presented as mean number of eosinophils and mast cells per 10^5^ μm^2^ and as area percent of CD3+ (T-cells) and CD20+ (B-cells). Statistical significance was assumed at *p < 0.017 and **p < 0.003. On day 14, three animals in the control group were treated topically with amitraz (Taktic^®^) at the recommended dose for animal welfare reasons.

Eosinophil numbers in the epidermis/superficial dermis and in the deep dermis were significantly depressed in the treatment group compared to the control group on day 7 and day 28. The number of mast cells did not differ between both groups. The CD3-positive T-cell areas were significantly reduced on day 28 after dexamethasone treatment. Although the mean CD20-positive B-cell area in the treatment group was lower than in the control group on day 28, there was no significant difference between the two groups (Figure 6).

## Discussion

Individual and breed differences in susceptibility to infestation with *P. ovis* and the related *Sarcoptes scabiei* (*S. scabiei*) mites have been associated with pathological changes and immune responses in various host species [2, 27–29]. The available studies suggest that the complex relationship between the host and the parasite at the skin interface results in a balance between the immune responses and clinical signs [30–32], yet many questions remain unclear.

The HF cattle breed is less susceptible to *P. ovis* infestation in comparison to BB cattle in the field [2, 33]. Given that the immune response is at least partially responsible for the lesion development, a possible reason for the difference of lesion development between BB and HF cattle could be related to different immune responses after *P. ovis* infestation. In the first animal study, the dermal immune response in BB cattle and HF cattle during an experimental *P. ovis* infestation was compared, in an attempt to identify a potential cause of the severe manifestation of psoroptic mange in the BB cattle breed compared to HF cattle.

The significant difference between the clinical indices of both breeds confirmed that BB cattle were more susceptible to the induced *P. ovis* infestation than HF. A previous study also showed that BB cattle were more susceptible to artificial *P. ovis* infestation than HF cattle, but the differences were not significant [2], which may be due to the small sample size. Apart from these quantitative differences, the clinical picture and histological changes in HF cattle were similar to BB cattle and were characterized by erythematous scaly papules and plaque formation and infiltration of inflammatory cells into the epidermis and dermis. Significant influxes of eosinophils in the epidermis/superficial dermis and the deep dermis were detected in comparison with the pre-infestation in BB cattle from 3 wpi onwards, but in HF cattle only at 5 wpi. Moreover, the number of eosinophils in BB cattle was significantly higher and increased steadily until the end of the experiment, whereas eosinophils in HF cattle peaked at 5 wpi and started to decline thereafter. However, the epidermal differentiation complex (EDC) genes, such as filaggrin, loricrin and involucrin were significantly upregulated in HF cattle, while the transcription of filaggrin and loricrin was slightly downregulated (NS) in BB cattle. Previous studies suggested that EDC genes could be down-regulated through the suppressive action of Th2 cytokines, such as IL-4 and IL-13 [34, 35]. Together with the more pronounced cutaneous eosinophilia and higher IL-4 levels at 6 wpi in BB cattle, this suggests that a Th2-type immune response is underlying the more severe manifestation in the BB breed. Similarly, a (pro-)inflammatory response, evolving into a Th2-type immune response was previously observed in *P. ovis* infestations in sheep [6, 36]. In sheep, the maximal number of eosinophils was observed at 9 wpi [36]. Possibly, differences between the two cattle breeds may become more pronounced later in the infestation.

The CD3-positive T-cell area in HF cattle was significantly higher than in BB cattle. Since no further T-lymphocyte markers were used in our experiment, the T-cell population could not be subtyped. We hypothesized that a (proportion of) the larger population of T lymphocytes in HF cattle could be regulatory T-cells that are able to suppress the immune responses [37]. However, this hypothesis was not supported by the transcription levels of IL-10 and Foxp3, which were not significantly different between the two breeds. An increased Th2/Th17 immune response was associated with increased susceptibility to *S. scabiei* infestations in humans and pigs [27, 29, 38] and in BB cattle naturally infested by *P. ovis* [19]. Although IL-17 levels were not significantly different within both breeds in our study, the expression of IL-17 was slightly increased in BB cattle and was decreased in HF cattle in comparison with pre-infestation. This is similar to the results of Sarre et al. [19]. Previous work in pigs with *S. scabiei* infestations showed an upregulation of IL-17 from 4 wpi onwards, whereas the peak expression was observed at 8 wpi [29]. Also, an increased IL-17 was detected at 15 wpi [38]. These results suggest that the Th17 immune response is related to the infestation time. Therefore, it would be interesting to investigate whether or not IL-17 transcription would further diverge between BB cattle and HF cattle when the infestation time is extended.

Since the higher susceptibility of BB cattle was shown to be associated with a stronger Th2-type response, our second objective was to investigate the causal relationship between the host’s immune response and the development of skin lesions. Treatment of BB cattle with dexamethasone induced a profound suppression of lesion development, but no significant reduction in mite numbers compared to the control group. Although in the study of Huntley et al. [11], anti-inflammatory treatment of sheep with Cyclosporin A led to significantly depressed lesion development and almost totally reduced mite numbers compared to the normal course of infection, previous studies showed that there is no strong relationship between the clinical index and mite numbers in cattle [2, 33]. Skin healing was accompanied by significantly reduced eosinophils and T-lymphocytes in the skin biopsies, which is similar with observations in sheep scab [11, 39]. In sheep, no difference was observed in the population of mast cells/basophils between control animals and animals treated with an anti-inflammatory drug [11], which is in accordance with the results of our study. Clearly, an inflammatory response is related to the development of psoroptic mange lesions in both sheep and cattle, but mast cells do not seem to be important in this process in cattle.

## Conclusions

The development of skin lesions in psoroptic mange in cattle is associated with the host immune response, as lesions development was reversed by anti-inflammatory treatment. The higher susceptibility of BB cattle compared to the HF breed appears to be due to a more pronounced Th2-type (and potentially Th-17) immune response in BB. A stronger T-cell response was observed in the more resistant HF breed, but it is as yet unclear with T-cell subtypes are involved. Identification of these T-cell subsets and extending the duration of the artificial infestations could further clarify the difference in the immune responses between both breeds. Local anti-inflammatory treatment could potentially be an alternative method for controlling the pathology caused by a *P. ovis* infestation.

## Supplementary information

**Additional file 1. Clinical appearance and close-up view of Belgian Blue (A, C) and Holstein–Friesian cattle (B, D) infested with** ***Psoroptes ovis*** **at 6** **weeks post-infestation, respectively.** The yellow circles indicate active lesions on the animals’ skin.

**Additional file 2. Representative histology of skin lesions at 6** **weeks post infestation with** ***Psoroptes ovis*** **in Belgian Blue cattle (A: ×100; B: ×400).** A: Parakeratosis (filled arrow), dermal oedema and disruption of collagen bundles (dashed line arrows). B: Intercellular oedema, disruption of intercellular bridges and hydeopic degeneration of cells in the epidermis (filled arrows)

**Additional file 3. The width of the epidermis at different time points in Belgian Blue (BB) and Holstein–Friesian (HF) cattle after artificial infestation with** ***Psoroptes ovis*** **(mean and individual values).** *p < 0.05, **p < 0.01, and ***p < 0.001. Only 7 BB cattle were included in the statistical analysis as from two animals a missing value was obtained at 3 and 4 weeks post-infestation.

## Data Availability

All data generated or analysed during this study are included in this published article and its Additional files.

## Abbreviations

BB: Belgium Blue
CI: clinical index
HF: Holstein–Friesian
IL-: interleukin-
*P. ovis*: *Psoroptes ovis*
*S. scabiei*: *Sarcoptes scabiei*
Wpi: weeks post infestation
Th: T-helper cell
