# Supplementary material for: Dermal immune responses against Psoroptes ovis in two cattle breeds and effects of anti-inflammatory dexamethasone treatment on the development of psoroptic mange
Source: Vet Res. 2021 Jan 4;52:1. doi: 10.1186/s13567-020-00874-x (PMC7784294; doi:10.1186/s13567-020-00874-x)
Supplement: Supplementary file 1 — Additional file 1. Clinical appearance and close-up view of Belgian Blue (A, C) and Holstein–Friesian cattle (B, D) infested with Psoroptes ovis at 6 weeks post-infestation, respectively. The yellow circles indicate active lesions on the animals’ skin. [file 13567_2020_874_MOESM1_ESM.docx]

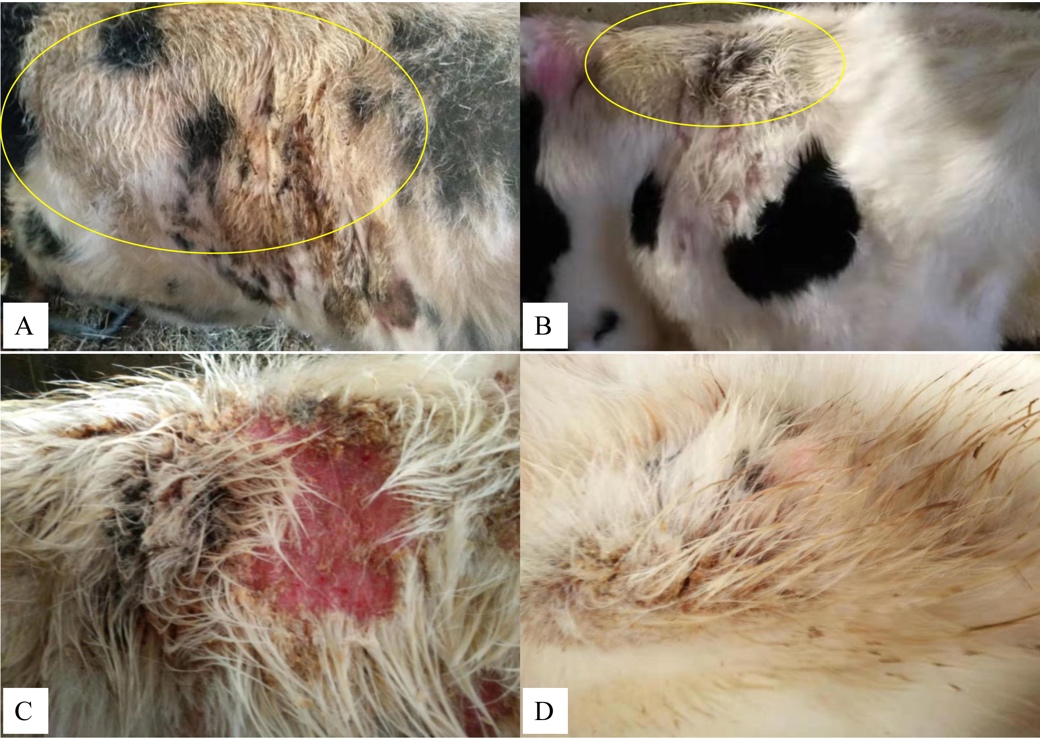


Additional file 1. Clinical appearance and close-up view of Belgian Blue (A, C) and Holstein-Friesian cattle (B, D) infested with *Psoroptes ovis* at 6 weeks post-infestation, respectively. The yellow circles indicate active lesions on the animals’ skin.
