# Supplementary material for: Dermal immune responses against Psoroptes ovis in two cattle breeds and effects of anti-inflammatory dexamethasone treatment on the development of psoroptic mange
Source: Vet Res. 2021 Jan 4;52:1. doi: 10.1186/s13567-020-00874-x (PMC7784294; doi:10.1186/s13567-020-00874-x)
Supplement: Supplementary file 2 — Additional file 2. Representative histology of skin lesions at 6 weeks post infestation with Psoroptes ovis in Belgian Blue cattle (A: ×100; B: ×400). A: Parakeratosis (filled arrow), dermal oedema and disruption of collagen bundles (dashed line arrows). B: Intercellular oedema, disruption of intercellular bridges and hydeopic degeneration of cells in the epidermis (filled arrows) [file 13567_2020_874_MOESM2_ESM.docx]

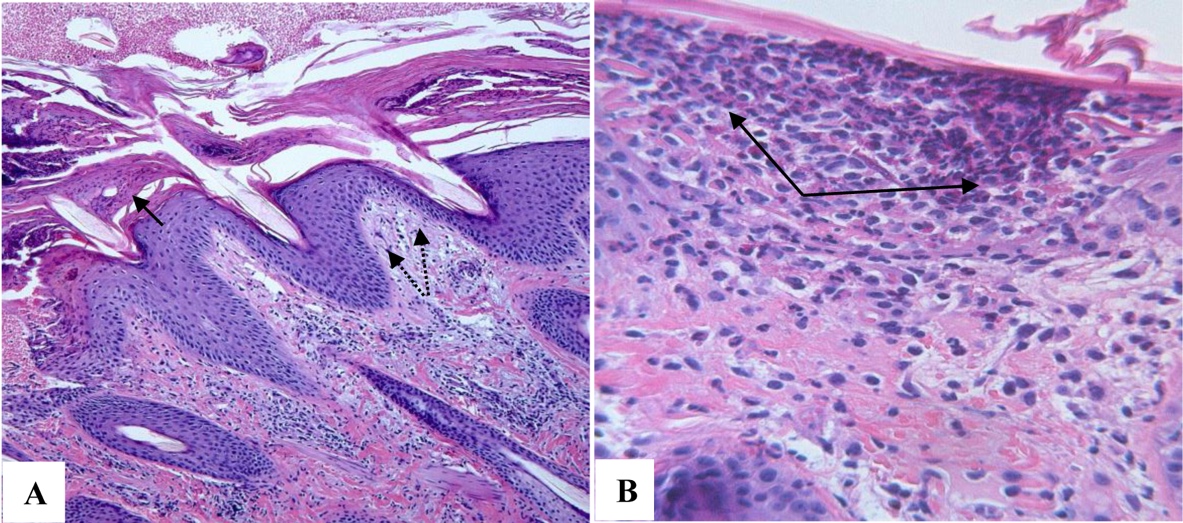


Additional file 2. Representative histology of skin lesions at 6 weeks post infestation with *Psoroptes ovis* in Belgian Blue cattle (A: ×100; B: ×400). A: Parakeratosis (filled arrow), dermal oedema and disruption of collagen bundles (dashed line arrows). B: Intercellular oedema, disruption of intercellular bridges and hydeopic degeneration of cells in the epidermis (filled arrows).
