# Supplementary material for: Dermal immune responses against Psoroptes ovis in two cattle breeds and effects of anti-inflammatory dexamethasone treatment on the development of psoroptic mange
Source: Vet Res. 2021 Jan 4;52:1. doi: 10.1186/s13567-020-00874-x (PMC7784294; doi:10.1186/s13567-020-00874-x)
Supplement: Supplementary file 3 — Additional file 3. The width of the epidermis at different time points in Belgian Blue (BB) and Holstein–Friesian (HF) cattle after artificial infestation with Psoroptes ovis (mean and individual values). *p < 0.05, **p < 0.01, and ***p < 0.001. Only 7 BB cattle were included in the statistical analysis as from two animals a missing value was obtained at 3 and 4 weeks post-infestation. [file 13567_2020_874_MOESM3_ESM.docx]

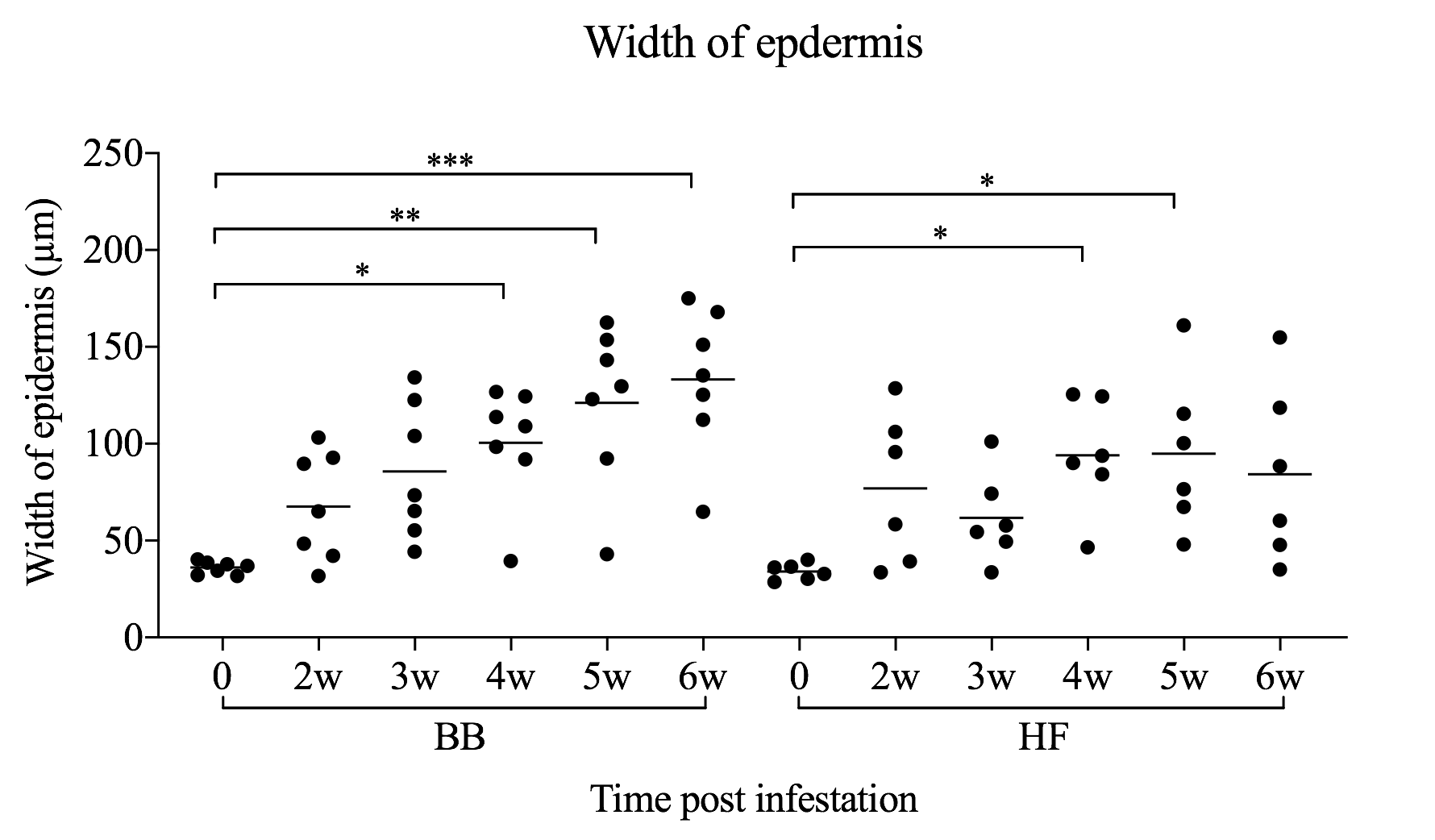


Additional file 3. The width of the epidermis at different time points in Belgian Blue (BB) and Holstein-Friesian (HF) cattle after artificial infestation with *Psoroptes ovis* (mean and individual values). *p<0.05, **p<0.01, and ***p<0.001. Only 7 BB cattle were included in the statistical analysis as from two animals a missing value was obtained at 3 and 4 weeks post-infestation.
